# Brain Tumor Segmentation from MRI Images Using Handcrafted Convolutional Neural Network

**DOI:** 10.3390/diagnostics13162650

**Published:** 2023-08-11

**Authors:** Faizan Ullah, Muhammad Nadeem, Mohammad Abrar, Muna Al-Razgan, Taha Alfakih, Farhan Amin, Abdu Salam

**Affiliations:** 1 Department of Computer Science, International Islamic University, Islamabad 44000, Pakistan; faizan.phdcs157@iiu.edu.pk (F.U.); nadeem@iiu.edu.pk (M.N.); 2 Department of Computer Science, Bacha Khan University, Charsadda 24420, Pakistan; abrar@bkuc.edu.pk; 3 Department of Software Engineering, College of Computer and Information Sciences, King Saud University, Riyadh 11345, Saudi Arabia; 4 Department of Information Systems, College of Computer and Information Sciences, King Saud University, Riyadh 11543, Saudi Arabia; talfakih@ksu.edu.sa; 5 Department of Information and Communication Engineering, Yeungnam University, Gyeongsan 38541, Republic of Korea; 6 Department of Computer Science, Abdul Wali Khan University, Mardan 23200, Pakistan

**Keywords:** optimization methods, computational approaches, brain tumor, feature fusion, handcrafted features, hybrid approach, segmentation

## Abstract

Brain tumor segmentation from magnetic resonance imaging (MRI) scans is critical for the diagnosis, treatment planning, and monitoring of therapeutic outcomes. Thus, this research introduces a novel hybrid approach that combines handcrafted features with convolutional neural networks (CNNs) to enhance the performance of brain tumor segmentation. In this study, handcrafted features were extracted from MRI scans that included intensity-based, texture-based, and shape-based features. In parallel, a unique CNN architecture was developed and trained to detect the features from the data automatically. The proposed hybrid method was combined with the handcrafted features and the features identified by CNN in different pathways to a new CNN. In this study, the Brain Tumor Segmentation (BraTS) challenge dataset was used to measure the performance using a variety of assessment measures, for instance, segmentation accuracy, dice score, sensitivity, and specificity. The achieved results showed that our proposed approach outperformed the traditional handcrafted feature-based and individual CNN-based methods used for brain tumor segmentation. In addition, the incorporation of handcrafted features enhanced the performance of CNN, yielding a more robust and generalizable solution. This research has significant potential for real-world clinical applications where precise and efficient brain tumor segmentation is essential. Future research directions include investigating alternative feature fusion techniques and incorporating additional imaging modalities to further improve the proposed method’s performance.

## 1. Introduction

Brain tumor segmentation is an essential process in medical image analysis, which aims to pinpoint the affected areas of the brain due to the presence of a tumor [1]. Diagnosis, therapy planning, disease progression monitoring, and precise and effective segmentation of brain tumors are crucial [2]. The complex nature of brain tumors and the differences between patients make manually identifying these tumors a tough and time-consuming job [3]. Brain tumors represent a heterogeneous group of intracranial neoplasms that affect both adults and children, posing significant challenges for diagnosis and treatment [4]. Magnetic resonance imaging (MRI) stands as the top choice for non-invasive brain tumor detection and assessment because of its exceptional resolution and outstanding contrast for soft tissues [5]. In many clinical tasks—for example, diagnosis, treatment planning, and patient monitoring—the accurate segmentation of brain tumors using MRI data is crucial [6]. The traditional segmentation methods are mainly based on handcrafted features, and these are designed based on domain knowledge [7]. The problem is that they are generally sensitive to variations in image intensity and hence require extensive manual tuning. Thus, the robustness and precision are very low [8]. Segmentation of brain tumor techniques is crucial for accurate diagnosis, monitoring of tumor progression, and treatment planning [9]. These techniques can be generally divided into three categories: manual brain tumor segmentation, semi-automatic, and fully automatic methods [10]. Manual segmentation of the brain is performed by radiologists or experts and involves the delineation of tumor regions on medical images using graphical tools [11]. This method can be accurate, but it is slow and takes a lot of work [12]. Furthermore, the increasing demand for medical imaging and the limited availability of expert radiologists make manual segmentation challenging to scale. Semi-automatic methods require minimal user intervention, often providing an initial contour or seed point to guide the segmentation process. These methods rely on algorithms such as region growing, which iteratively groups neighboring pixels with similar intensity values [13]; level-set methods, which evolve a contour based on geometric and image-based properties [14]; and active contours or snakes, which deform a curve or surface to minimize an energy function derived from image features [15]. Semi-automatic methods offer improved efficiency compared to manual methods; however, they still require user interaction, which can be time-consuming and may introduce variability. In fully automatic methods, tumors can be segmented without user interaction, such as machine learning (ML) and DL approaches. These techniques seek to increase the segmentation process’ effectiveness, consistency, and scalability [16]. Handcrafted feature-based methods involve extracting engineered features from images and training ML classifiers for tumor segmentation, while DL techniques such as CNN automatically learn hierarchical representations of the data to perform segmentation [17]. Fully automatic methods have demonstrated the potential for high precision and accuracy; however, they may require large, annotated datasets for training and can be computationally expensive. Recently, convolutional neural networks (CNNs) have emerged as a powerful resource for computer vision tasks, for instance, segmentation, etc. [9]. As compared to the traditional methods, CNNs have shown superior performance, especially in medical image segmentation, by introducing learning features from the data [10]. However, the integration of handcrafted features and CNNs for brain tumor segmentation has not been thoroughly investigated in the literature. From the literature, we identify that combining the handcrafted features and CNNs could lead to improving the overall performance by leveraging the strengths of both methods. Thus, based on this discussion, this study proposes a novel hybrid approach and suggests combining the handcrafted features and CNNs for brain tumor segmentation in MRI scans.

Briefly, our study contributes to the research on brain tumor segmentation by introducing a hybrid approach that combines handcrafted features with CNN to enhance the performance of brain tumor segmentation from MRI scans. The proposed hybrid model outperforms traditional handcrafted feature-based methods and individual CNN-based methods for brain tumor segmentation. In addition, it provides a more robust and generalizable solution with significant potential. The key contributions of this study are given below:

- Our proposed approach integrates various handcrafted features, for example, intensity, texture, and shape-based features and CNNs together to achieve high accuracy and robustness. In addition, the proposed approach has a better generalization capability for the unseen data.
- The efficiency of our proposed model is measured by comparing it with state-of-the-art segmentation models using standard benchmark datasets. The efficient results were measured based on various standard metrics, for instance, segmentation accuracy, Dice score, specificity, and sensitivity. The achieved results prove that our proposed model is highly efficient.
- This research has significant potential for real-world clinical applications where precise and efficient brain tumor segmentation is essential.

The rest of the paper is organized as follows: Section 2 presents a recent literature review on brain tumor segmentation techniques, examining the latest developments in the field, focusing on handcrafted feature-based methods, CNN-based methods, and hybrid approaches in medical imaging. Section 3 describes the proposed methodology, including data acquisition and pre-processing, handcrafted feature extraction, CNN architecture, and the hybrid approach for integrating handcrafted features and CNNs. Section 4 presents the experiments and results, discussing dataset description, evaluation metrics, comparative analysis, and performance discussion. In Section 5, this study concludes the paper’s limitations and future work.

## 2. Related Work

In recent years, extensive research has been carried out on brain tumor segmentation using handcrafted feature extraction techniques and deep learning (DL) approaches. In this section, we provide a detailed overview of related work in both areas.

### 2.1. Handcrafted Features-Based Methods

Medical image analysis has made extensive use of handcrafted feature-based techniques, including brain tumor segmentation. These techniques involve the segmentation of images using ML algorithms and the extraction of engineered features that define image qualities [18]. Handcrafted features are divided into three categories: intensity-based, texture-based, and shape-based. Intensity-based features capture the local intensity distribution within the image. These features include statistical measurements such as mean, median, standard deviation, and histogram-based metrics [19]. Intensity-based features are useful in differentiating between normal and abnormal tissue regions due to their distinct intensity profiles. Texture-based features describe the spatial arrangement of intensities and reflect the local patterns in the image. Common texture-based features include the gray-level co-occurrence matrix (GLCM), which captures the frequency of specific pixel value combinations at certain spatial relationships [20]; local binary patterns (LBP), which encode the relationship between a pixel and its neighbors [21]; and Gabor filters, which analyze the frequency and orientation information in images [22]. Texture features can be valuable for characterizing the heterogeneity and complexity of tumor regions. Shape-based features capture the geometric properties of the tumor region, providing information about the tumor’s size, shape, and boundary irregularities. Examples of shape-based features include area, perimeter, compactness, and various moments [23]. These features can help differentiate tumors from surrounding tissues based on their distinct morphological characteristics.

ML algorithms, such as random forests (RF), support vector machines (SVM), and k-nearest neighbors (k-NN), are trained for segmentation tasks after handcrafted features are extracted [24]. Despite the success of handcrafted feature-based methods in various medical image segmentation tasks, these methods often require extensive manual tuning and are sensitive to variations in image intensity, limiting their robustness and precision [25]. Additionally, the reliance on manually engineered features can lead to a lack of adaptability to diverse imaging conditions and tumor appearances. Therefore, there is a need for more robust and versatile approaches to tumor segmentation.

### 2.2. Convolutional Neural Network-Based Methods

CNN has revolutionized the field of image recognition and segmentation by automatically learning features from the data, making them more robust to variations and alleviating the need for manual feature engineering [17]. CNNs are composed of multiple layers, including convolutional, pooling, and fully connected layers, that use nonlinear transformations to learn hierarchical representations of the input data [26]. This allows CNNs to effectively capture complex patterns and structures within images, leading to improved performance in various image analysis tasks.

Several CNN architectures have been proposed in the context of brain tumor segmentation to address the challenges faced by the heterogeneity and complexity of brain tumors. Some of the most prominent architectures include U-Net, V-Net, and DeepMedic [27,28,29]. The accurate localization of tumor boundaries is made possible by the symmetric encoder-decoder architecture known as U-Net, which uses skip connections to merge low-level and high-level data. V-Net extends the U-Net architecture to 3D medical images and incorporates a volumetric loss function for improved segmentation performance. DeepMedic employs a multi-scale approach with parallel processing of image patches at different resolutions to capture both global and local contextual information. The study in [30] aimed to accurately segment brain tumors from MRI scans using a 3D nnU-Net model enhanced with domain knowledge from a senior radiologist. The approach improved the model’s performance and achieved high Dice scores for the validation and test sets. The approach was validated on hold-out testing data, including pediatric and sub-Saharan African patient populations, demonstrating high generalization capabilities.

These CNN architectures have demonstrated superior performance in brain tumor segmentation compared to traditional methods by learning context-aware features that capture both local and global information [31]. Additionally, CNN-based methods are more robust to intensity variations and can adapt to diverse imaging conditions and tumor appearances, making them a promising approach for this task.

Despite the success, CNNs usually need large, annotated datasets for training, which can be challenging to obtain in the medical domain due to the limited availability of expert annotations and the time-consuming nature of manual segmentation [32]. Furthermore, CNNs can be computationally expensive, particularly for large 3D medical images, and may lack interpretability due to their black-box nature.

To overcome these limitations, researchers have explored various strategies, such as transfer learning, data augmentation, and incorporating domain knowledge through the integration of handcrafted features. These approaches aim to leverage the strengths of both handcrafted feature-based methods and CNNs to improve the robustness, precision, and interpretability of brain tumor segmentation techniques.

### 2.3. Hybrid Approaches in Medical Imaging

Hybrid approaches aim to combine the strengths of handcrafted features and DL techniques to increase the performance of medical image segmentation tasks, taking advantage of domain knowledge and automated feature learning [33]. Several hybrid approaches have been proposed for various medical imaging applications, including lung nodule detection, breast cancer segmentation, and retinal vessel segmentation [34,35,36].

These hybrid approaches often involve integrating handcrafted features at different levels of the CNN architecture, such as input channels, feature maps, or decision levels [33]. Several strategies have been proposed for incorporating handcrafted features into DL models. One approach is to concatenate handcrafted features with deep features before the classification layer, which allows the model to leverage both feature types during the decision-making process [36]. Another approach involves injecting handcrafted features into intermediate layers of the CNN, enabling the network to learn more complex, higher-level representations that integrate domain knowledge [37]. Multi-stream architectures, which process handcrafted and deep features in parallel, have also been proposed to encourage complementary learning and robust feature representations [38].

These hybrid approaches have demonstrated an improved performance compared to individually handcrafted features or CNN-based methods in various medical imaging tasks. By combining the advantages of both techniques, hybrid models can capitalize on the domain knowledge provided by handcrafted features while benefiting from the automatic feature learning capabilities of CNNs.

Table 1 summarizes a comparison of brain tumor segmentation techniques, including handcrafted feature methods, CNN-based, and hybrid approaches. The evaluation of the relevant literature emphasizes the limitations of handcrafted feature-based methods and the potential of CNN-based methods for tumor segmentation. However, the integration of handcrafted features and CNNs has not been thoroughly investigated for brain tumor segmentation. A hybrid method that combines the strengths of each approach could lead to improved performance in this task, offering a promising avenue for future research and development in the field of medical image analysis.

### 2.4. Data Augmentation Techniques

The literature includes an extensive collection of data augmentation in brain MRI. Different techniques were applied to the MRI such as translation, noise addition, rotation, and shearing to increase the size of the dataset as well the performance of tumor segmentation. Khan et al. [39] applied the noise addition to and shearing methods to increase the size of the dataset and improved the accuracy of the classification and tumor segmentation. Similarly, Dufumier et al. [40] applied rotation, random cropping, noise addition, translation, and blurring to increase the dataset size and performance in the prediction of age, and sex classification. Different studies used elastic deformation, rotation, and scaling to improve tumor segmentation and accuracy at the same time [41]. These techniques are common due to their simplicity and performance. In addition to these techniques, the researchers also generated synthetic images to perform a specific task. The most common method of image generation is the mix-up, where the patches from two random images are combined to generate the new image. In all these applications, the researchers used different datasets and different numbers of images. Similarly, everyone used a different network architecture. Thus, every researcher presented the results performance based on their selected techniques. In this article, after careful evaluation of the literature, the common techniques are used. These techniques are presented in Table 2. Furthermore, Nelapa et al. [42] provided a comprehensive survey of the data augmentation that can be used for further details.

## 3. Methodology

**Figure 1:**
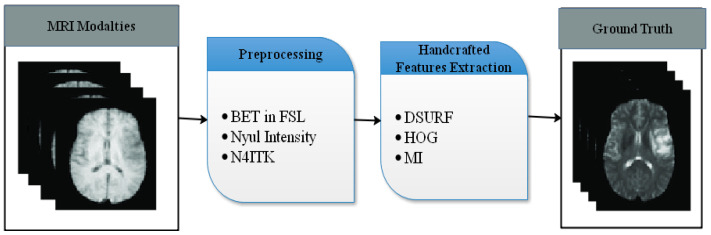
Handcrafted feature extraction.

In this section, we describe the proposed methodology for brain tumor segmentation using a fusion of handcrafted features and CNN. The proposed method consists of two feature pathways for handcrafted and CNN. Pre-processing and data augmentation are also applied. An overview of the proposed methodology is presented in Figure 1.

### 3.1. Data Acquisition and Preprocessing

The Brain Tumor Segmentation (BraTS) 2018 dataset, which is freely available, provided the MRI scans used in this study [43]. BraTS provides multi-institutional, multi-scanner, and multi-protocol pre-operative scans of patients with brain tumors. The dataset contains four different MRI modulates for each patient: T1-weighted, T1-weighted post-contrast (T1C), T2-weighted, and T2-FLAIR. These sequences provide complementary information about the tumor and its surroundings, allowing for a more comprehensive analysis of the tumor’s characteristics. Table 3 presents the dataset’s distribution in terms of the number of patients with gliomas and their respective tumor classifications.

Before feeding the MRI scans into the proposed model, several pre-processing techniques were applied to standardize the input MRI slice. To achieve spatial alignment between various sequences, the MRI scans were co-registered to a common reference space using a rigid registration technique [44]. The skull and other non-brain tissues were removed from the MRI scans using a skull stripping algorithm, such as the Brain Extraction Tool (BET) in FSL [45], to reduce noise and computational complexity. The intensity values of the MRI scans were normalized to a standard range of 0 and 1 to minimize the effects of intensity variations across different scanners and protocols [46]. MRI scans often suffer from intensity inhomogeneity due to the presence of a biased field. The N4ITK algorithm was used to correct the bias field and achieve uniform intensity distributions across the images [47].

### 3.2. Handcrafted Feature Extraction

The proposed hybrid approach for brain tumor segmentation combines handcrafted features and CNNs. In this section, the handcrafted feature extraction process is described, which includes the Dense SURF (DSURF) descriptor and Histogram of Oriented Gradients (HOG) features shown in Figure 1.

#### 3.2.1. DSURF Descriptor

The Speeded Up Robust Features (SURF) is a variation that includes the DSURF descriptor, which is utilized for both feature point detection and description [48]. DSURF selects dense feature points situated closely together along a grid with a specific step size, resulting in a significant feature gain compared to SURF when prior knowledge is limited. Each key point is assigned a feature descriptor, and the SURF descriptor can have 64 or 128 dimensions. After identifying the key point, an orientation is defined in a circular region around the key point, which is then aligned to derive the SURF descriptor. The DSURF descriptor extraction is given as follows:

Grid Creation:

(1) G(x,y)=(x×s,y×s)

where *x* and *y* are both integers and *s* are a specific step size.

Feature detection (Matrix *H*):

(2) H=[Lxx(x,σ)Lxy(x,σ)Lxy(x,σ)Lyy(x,σ)]

where *σ* represents a standard deviation value.

Orientation assignment:

(3) $$\theta =\arctan(\frac{\sum W(x,y)\times Lx(x,y)}{\sum W(x,y)\times Ly(x,y)})$$

SURF:

(4) D=[∑Lx,∑Ly,∑|Lx|,∑|Ly|] for each sub-region.

#### 3.2.2. HOG Features

HOG features have been widely employed in a variety of applications, including pedestrian recognition, object identification, image registration, and medical image categorization [49]. HOG calculates the number of times an oriented gradient appears in a certain area of an image, capturing edge information that may be used for categorization. The image is divided into small, contiguous cells, and the edge orientations or HOG directions for each cell are determined. The resulting histograms are combined to form the descriptor. Using the following equations, gradients are computed:

(5) $$Gx=\frac{\partial f(x,y)}{\partial x}=\frac{f(x+1,y)-f(x-1,y)}{\left(x+1)-(x-1\right)}$$

(6) $$Gy=\frac{\partial f(x,y)}{\partial y}=\frac{f(x,y+1)-f(x,y-1)}{\left(y+1)-(y-1\right)}$$

Every block in the HOG process generates the density of its intensity gradients. A feature vector represents the information received from distinct parts of an image.

### 3.3. CNN Architecture

The suggested CNN architecture is based on the U-Net architecture and is intended to segregate brain tumors. The architecture is made up of an encoding path that collects context information and a decoding path that allows for exact localization [27]. Table 4 shows the architecture of the proposed CNN.

#### Training Procedure and Hyperparameters

The proposed CNN was trained with a mix of cross-entropy and Dice coefficient losses. As shown in Equation (7), Cross Entropy Loss is calculated by Equation (9):

(7) $$Entropy=-\underset{y=i}{\sum }yi\times \log(f(x))i$$

The Cross-Entropy Loss function measures the dissimilarity between the predicted probability distribution (*f*(*x*)) and the true distribution (*y*), while the Dice coefficient is calculated below:

(8) $$Dice\;Coefficient=\frac{1-2\times \sum_{yi}\times f(x)+\varepsilon }{\sum_{yi}+\sum_{ji}\times f(x)+\varepsilon }$$

(9) Loss=Entropy+(1−Dice Coefficient)

The training dataset is divided into mini-batches, and the weights are updated with momentum using the stochastic gradient descent (SGD) optimization algorithm. Key hyperparameters for the training process are provided in Table 5.

To prevent overfitting, the model is trained for 100 epochs and measures its performance on a validation set. Early stopping is used to end training when the validation loss does not improve after a certain number of epochs. The proposed methodology leverages a CNN architecture inspired by U-Net, which is trained using a combination of cross-entropy and Dice coefficient loss. The proposed model is trained over the SGD optimizer with momentum and early halting. By gathering both local and global information from the input MRI slices, this architecture seeks to achieve accurate and exact brain tumor segmentation.

### 3.4. Integrating Handcrafted Features and CNN

**Figure 2:**
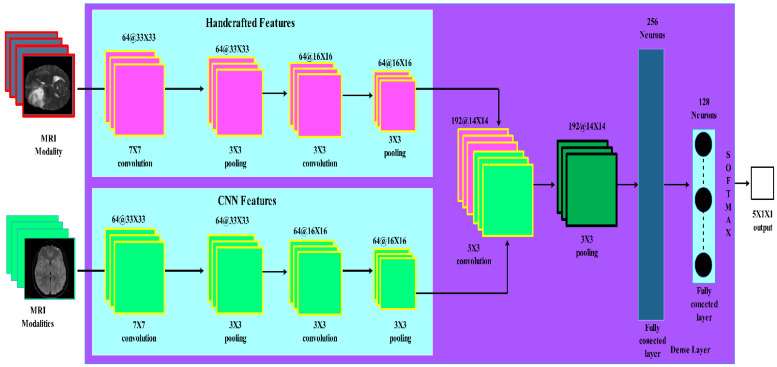
Proposed hybrid approach with multiple pathways.

The hybrid approach aims to leverage the strengths of both handcrafted features and CNN for improved brain tumor segmentation. In this approach, handcrafted features are integrated into the CNN architecture to create a more robust and accurate model. The proposed model consists of two input channels for handcrafted features and CNN features. In the next stage, a feature map is calculated, and handcrafted features are fused with feature maps extracted from intermediate layers of the CNN. In the next stage, handcrafted features are concatenated with the output of the last CNN layer before the final classifier as shown in Figure 2.

These strategies include input channel fusion, feature map fusion, and decision level fusion. By combining handcrafted features with CNN features, the model can capture both low-level and high-level information to improve segmentation performance.

#### Fine-Tuning the CNN

After integrating handcrafted features, CNN is fine-tuned to adapt to the new input representation. The fine-tuning process involves updating the weights of the model by minimizing the same loss function as used in the initial training Equation (3).

The hyperparameters for fine-tuning are like those used in the initial training The learning rate is minimized by a factor of 10 to ensure that the fine-tuning process does not drastically change the learned features. The fine-tuning process is performed for a smaller number of epochs (e.g., 50) to avoid overfitting, as the model has already been trained on the dataset. The proposed hybrid approach integrates handcrafted features with the CNN architecture to create a more robust and accurate model for brain tumor segmentation. Different feature fusion strategies are proposed for combining handcrafted and CNN features at various levels of the architecture. CNN is then fine-tuned to adapt to the new input representation, with a reduced learning rate and fewer epochs to prevent overfitting. This hybrid approach aims to leverage the strengths of both handcrafted features and CNNs for improved performance in brain tumor segmentation tasks.

### 3.5. Evaluation Metrics

To assess the performance of this study, various evaluation techniques are used, including segmentation accuracy, Dice score, specificity, and sensitivity. These metrics provide a comprehensive assessment of the segmentation performance, considering various aspects such as overlap, false positives, and false negatives.

#### 3.5.1. Segmentation Accuracy

Segmentation accuracy is a widely used metric in image segmentation tasks. In terms of the total number of pixels in the image, it calculates the percentage of properly identified pixels, mathematically defined as below:

(10) $$Accuracy=\frac{\left(TP+TN\right)}{\left(TP+TN+FP+FN\right)}$$

In Equation (10), True Positive (TP), True Negative (TN), False Positive (FP), and False Negative (FN) stand for the respective counts of true positives, true negatives, false positives, and false negatives. While segmentation accuracy provides an overall assessment of the segmentation performance, it may be misleading in cases of class imbalance, where one class is significantly more prevalent than the others.

#### 3.5.2. Dice Score

The Dice score, commonly referred to as the Dice similarity coefficient, is a well-liked statistic for determining how much the projected and actual segmentation masks coincide. It is defined as below:

(11) $$Dice\;Score=\frac{2\times TP}{2\times TP+FP+FN}$$

The Dice score goes from 0 to 1, with 0 denoting complete overlap and 1 denoting no overlap at all. This metric is particularly useful in medical image segmentation, as it accounts for both false positives and false negatives and is less sensitive to class imbalance compared to segmentation accuracy.

#### 3.5.3. Sensitivity and Specificity

In medical image analysis, sensitivity and specificity measurements are frequently used metrics to assess the effectiveness of binary classification tasks. Sensitivity, also known as the true positive rate or recall, is a measurement of the proportion of positive cases containing true positives. Specificity, also known as the true negative rate, is the calculation of the number of true negatives in truly negative cases. These sensitivity and specificity metrics are defined as:

(12) $$Sensitivity=\frac{TP}{\left(TP+FN\right)}$$

(13) $$Specificity=\frac{TN}{\left(TN+FP\right)}$$

Sensitivity and specificity provide complementary information about the segmentation performance, as sensitivity focuses on the ability of the method to correctly identify positive cases (like tumor regions), while specificity focuses on the ability to correctly identify negative cases in non-tumor regions. By considering both sensitivity and specificity, a more comprehensive assessment of the segmentation performance can be obtained.

### 3.6. Experimental Setup

In this article, we harnessed the power of Google Colab to set up and conduct experiments using Python 3, taking full advantage of its default GPU setting. Google Colab provides an excellent platform for machine learning and deep learning tasks, and its integration with Python 3 makes it an attractive choice for researchers, developers, and students alike.

To build and train convolutional neural networks (CNNs), we leveraged the capabilities of TensorFlow, one of the most widely used and well-documented deep learning libraries. TensorFlow’s intuitive interface and extensive community support enabled us to design complex neural network architectures for various computer vision tasks.

## 4. Results and Discussion

This study proposed a hybrid approach that combines handcrafted features and CNN. The integration of handcrafted features with CNN features in our proposed hybrid approach led to improved segmentation performance. This approach allowed us to leverage the strengths of both handcrafted features and CNN for more accurate and robust tumor segmentation. The fine-tuning of the CNN on the integrated features further improved the performance of our approach.

### 4.1. Brain Tumor Segmentation Challenge Dataset

In this section, we evaluate our approach to the Brain Tumor Segmentation Challenge (BraTS) 2018 dataset. The BraTS dataset includes multi-modal MRI images from patients with brain tumors, containing four MRI modalities: T1-weighted (T1), T1-weighted post-contrast (T1-Gd), T2-weighted (T2), and fluid-attenuated inversion recovery (FLAIR). There are 285 patients in the dataset, comprising 200 scans for training and 85 scans for testing, which is a traditional 70:30 ratio splits.

Each MRI scan has 155 axial slices with a resolution of 240 × 240 pixels. The BraTS dataset includes ground truth labels for three tumor sub-regions: the enhancing tumor (ET), the tumor core (TC), and the whole tumor (WT). Predicting the voxel-wise labels for these sub-regions in the MRI images is part of the segmentation task. The given ground truth labels enable a quantitative evaluation of the proposed technique, with conventional metrics like Dice score, sensitivity, and specificity used to measure performance.

### 4.2. Data Augmentation Techniques

To enhance our proposed model’s performance to generalize and prevent overfitting caused by the dataset’s limited size, on the training data, we use data augmentation methods. Random rotation, scaling, and horizontal flipping of the MRI images. Additionally, random intensity shifts and contrast normalization are performed to account for intensity variations between patients.

Data augmentation approaches enhance the heterogeneity in the training dataset, allowing the model to learn more robust features and perform better on unobserved data. The combination of these techniques ensures that the model can handle potential variations in the input data, such as differences in imaging protocols, scanner types, and patient populations.

### 4.3. Performance of Handcrafted Feature-Based Methods

The performance of various handcrafted feature-based methods is evaluated, including MI features, HOG features, and SURF features. The results are summarized in Table 6.

### 4.4. Performance of CNN-Based Methods

The performance of various CNN-based methods is evaluated, including U-Net, V-Net, and DeepMedic. The performance of U-Net is comparatively better than that of the traditional CNN due to its distinctive features, i.e., skip connection. The results are summarized in Table 7.

### 4.5. Performance of the Proposed Hybrid Approach

The performance of the proposed hybrid approach, which combines handcrafted features and the proposed CNN, is evaluated. The results are summarized in Table 8.

The comparative analysis shows that the proposed hybrid approach outperforms both handcrafted feature-based methods and CNN-based methods in terms of segmentation accuracy, Dice score, sensitivity, and specificity. This demonstrates the effectiveness of the hybrid approach in leveraging the strengths of handcrafted features and DL techniques for brain tumor segmentation.

### 4.6. Impact of Handcrafted Features on CNN Performance

The integration of handcrafted features into the CNN model proved to have a positive impact on the segmentation performance, as demonstrated in Table 9. The hybrid technique proposed outperformed CNN-based methods in terms of segmentation precision, Dice score, sensitivity, and specificity. This enhancement is due to the complementary character of the custom-designed and CNN-learned features.

The combination of handcrafted features and CNN-based features allows the model to capture a wide range of information, increasing its ability to handle these variations.

In addition, the hybrid approach demonstrated better generalization capabilities compared to individual handcrafted feature-based and CNN-based methods. By leveraging the strengths of both types of features, the model can generalize well to unseen data, making it a promising solution for real-world applications in clinical settings.

This study’s findings demonstrate the feasibility of the proposed hybrid method for accurate and robust brain tumor segmentation. Future research could investigate the effect of various feature fusion strategies and fine-tuning techniques on the hybrid model’s performance. Furthermore, the integration of other handcrafted features or advanced DL techniques, such as attention mechanisms, could be explored to enhance the segmentation performance even further.

## 5. Conclusions

In this research, a hybrid approach for brain tumor segmentation that combines handcrafted features and CNNs is presented. The methodology involved data acquisition and pre-processing, feature extraction, CNN architecture, and the integration of handcrafted features and CNNs. The proposed hybrid approach demonstrated a superior performance compared to individual handcrafted feature-based and CNN-based methods. The integration of handcrafted features and CNNs led to improved segmentation accuracy and robustness, as well as better generalization capabilities for unseen data. Despite the promising results, the proposed hybrid approach has some limitations. One limitation is the complexity of integrating handcrafted features and CNNs, which can require extensive tuning to achieve optimal performance. Moreover, the approach still relies on the availability of large, annotated datasets for training, which can be challenging to obtain in the medical domain. Future work could address these limitations by investigating the impact of different feature fusion strategies, fine-tuning techniques, and the integration of advanced DL techniques, such as attention mechanisms or domain adaptation. Furthermore, exploring the use of transfer learning and unsupervised or semi-supervised learning methods could help overcome the challenge of limited annotated datasets and improve the generalization capabilities of the model across different medical imaging datasets and modalities.

## Figures and Tables

**Table 1:** Comparison of brain tumor segmentation techniques.

Technique	Advantages	Disadvantages
Handcrafted Features	Domain knowledge	Sensitive to intensity variations
	Compatibility with traditional ML	Require manual tuning
CNN-based Methods	Automatic feature learning	Require large, annotated datasets
	Robustness to intensity variations	Computationally expensive
	High precision and accuracy	May lack interpretability
Hybrid Approaches	Strengths of both handcrafted and CNN features	The complexity of the integration strategy
	Improved performance	Large, annotated datasets and tuning
	Potential for increased interpretability	

**Table 2:** Data augmentation techniques.

Technique	Description
Rotation	Randomly rotate the MRI scans by ±15 degrees
Scaling	Randomly scale the MRI scans by a factor between 0.8 and 1.2
Horizontal Flip	Randomly flip the MRI scans horizontally with a probability of 0.5
Elastic Deformation	Apply random elastic deformation to the MRI scans with a Gaussian filter of σ = 4.0
Intensity Shift	Randomly shift the intensity of the MRI scans by a factor between −0.1 and 0.1
Contrast Normalization	Normalize the contrast of the MRI scans by histogram equalization

**Table 3:** Distribution of the BraTS 2018 dataset.

Tumor Grade	Number of Patients
High-Grade	210
Low-Grade	75
Total	285

**Table 4:** Proposed CNN architecture.

Layer Type	Output Size	Activation Function
Input	256 × 256 × 4	-
Convolution	128 × 128 × 64	ReLU
Convolution	32 × 32 × 128	ReLU
Max Pooling	16 × 16 × 128	-
Convolution	8 × 8 × 256	ReLU
Max Pooling	4 × 4 × 256	-
Convolution	2 × 2 × 512	ReLU
Up-sampling	4 × 4 × 512	-
Convolution	8 × 8 × 256	ReLU
Up-sampling	16 × 16 × 256	-
Convolution	32 × 32 × 128	ReLU
Up-sampling	64 × 64 × 128	-
Convolution	128 × 128 × 64	ReLU
Up-sampling	256 × 256 × 64	-
Convolution	256 × 256 × 4	Softmax

**Table 5:** Training hyperparameters.

Hyperparameters	Value
Momentum	0.9
Learning Rate	0.0010
Weight Decay	0.0005
Batch Size	16
Number of Epochs	100
Loss Function	Equation (3)
Optimizer	SGD

**Table 6:** Performance of handcrafted feature-based methods.

Method	Accuracy	Dice Score	Specificity	Sensitivity
MI	0.75	0.65	0.72	0.77
HOG	0.80	0.70	0.76	0.82
SURF	0.82	0.74	0.79	0.84

**Table 7:** Performance of CNN-based methods.

Method	Accuracy	Dice Score	Specificity	Sensitivity
U-Net	0.90	0.85	0.88	0.91
V-Net	0.92	0.88	0.90	0.93
DeepMedic	0.93	0.89	0.91	0.94

**Table 8:** Performance of the proposed hybrid approach.

Method	Accuracy	Dice Score	Sensitivity	Specificity
Proposed hybrid approach	0.95	0.91	0.93	0.96
Asodekar et al. [23]	0.82	0.74	0.79	0.84
Ronneberger et al. [27]	0.90	0.85	0.88	0.91
Milletari et al. [28]	0.92	0.88	0.90	0.93
Kamnitsas et al. [29]	0.93	0.89	0.91	0.94
Raza et al. [34]	0.94	0.90	0.92	0.95

**Table 9:** Performance comparison between CNN and hybrid approach.

Method	Accuracy	Dice	Specificity	Sensitivity
Handcrafted	0.84	0.72	0.78	0.89
CNN	0.88	0.79	0.84	0.92
Proposed hybrid approach	0.95	0.91	0.96	0.93

## Data Availability

Datasets analyzed during the current study are available on the BraTS website [43].
